# Investigation on the Prevalence of Canine Microfilaremia in Thailand Using a Novel Microfluidic Device in Combination with Real-Time PCR

**DOI:** 10.3390/vetsci8030039

**Published:** 2021-02-28

**Authors:** Sumas Loymek, Achinya Phuakrod, Kati Zaelai, Witsaroot Sripumkhai, Prapakorn Vongjaroensanti, Sirichit Wongkamchai

**Affiliations:** 1 Office of Disease Prevention and Control Region 12 Songkhla, Department of Disease Control, Ministry of Public Health, Songkhla 90000, Thailand; sumatloimek@gmail.com; 2 Department of Microbiology, Faculty of Medicine Siriraj Hospital, Mahidol University, Bangkok 10700, Thailand; lek.achinya@gmail.com; 3 Department of Parasitology, Faculty of Medicine Siriraj Hospital, Mahidol University, Bangkok 10700, Thailand; katiz54@hotmail.com (K.Z.); prapagorn.nin@gmail.com (P.V.); 4 Thai Microelectronic Center, National Electronics and Computer Technology Center, Thailand Science Park, Pathumthani 12120, Thailand; witsaroot.sripumkhai@nectec.or.th

**Keywords:** *Brugia malayi*, *Brugia pahangi*, *Dirofilaria immitis*, HRM real time PCR, microfluidic

## Abstract

We conducted a survey of canine microfilaraemia in 768 dogs in Chanthaburi, Samut Sakhon, and Narathiwat provinces of Thailand using a novel semi-automated, microfluidic device that is easy and rapid to perform. Microfilariae species were identified using High Resolution Melting real-time PCR (HRM real-time PCR). The prevalence of canine microfilaremia was 16.2% (45/278) in Chanthaburi and 5.5% (12/217) in Samut Sakhon. The prevalence of canine microfilaremia in Narathiwat was 22.7% (67/273). *Brugia pahangi* and *Dirofilaria immitis* were the predominant species of filariae found in the infected dogs from Chanthaburi and Narathiwat, respectively. The low prevalence of canine microfilaremia of Samut Sakhon may reflect the success of the Soi Dog foundation’s efforts and the establishment of veterinary control programs. An effective disease control and prevention strategies is needed in Chanthaburi and Narathiwat to reduce the risks of zoonotic transmission of the parasites. An appropriate drug treatment should be given to infected dogs and prophylactic drugs are suggested to be given to dogs age ≤1-year-old to prevent filarial infection. The novel microfluidic device could be implemented for surveillance of filariae infection in other animals.

## 1. Introduction

Filariasis is a disease caused by nematodes in the Filarioidea superfamily [1,2]. Human infections with the filariae of animals, referred to as zoonotic filariasis, occur worldwide. *Dirofilaria immitis*, *Dirofilaria repens*, *Brugia malayi*, and *Brugia pahangi* have been reported as important zoonotic filariae from dogs and cats [2,3,4,5,6]. In Thailand, human cases of zoonotic filariasis caused by *Dirofilaria* spp. and *B. pahangi* have also been reported [7,8,9]. In another report from Thailand, two cases of brugian filariasis were reported in Rayong province, with suspected zoonotic origin. *Brugia pahangi* was the infective agent and dogs are suspected sources of the infection [10].

Filarial infection in dogs in Thailand has been reported for the past 30 years. In 1987, the high prevalence of canine filarial infection (45.67%) had been reported from Chiang Mai [11]. A study in Bangkok found a 13.9% filarial infectivity rate in stray dogs [12] whereas Kamyingkird et al. reported a prevalence of 24.1% of *D. immitis* infection in stray dogs in Songkla and Satun [13]. All of these studies were performed in urban cities such as Bangkok, Chiang-Mai, and Songkhla where *Culex* spp., which is the main vector of *Dirofilaria*, is a common mosquito species in urban areas that “thrives and proliferates excessively in crowed city areas” [14], *D. immitis* was the only species of filariae reported in these studies.

Climate change, extended ranges for vectors and infected animal reservoirs, increasing urbanization, and outdoor recreational activities are among the factors that have broadened the profile of filariasis-affected individuals [15]. The awareness of the potential zoonotic filarial infections, the knowledge of the incidence, and the impact of zoonotic parasites in animal populations are crucial points to prevent the infection in people and other animals and to control the spread of the filariae.

In the present study, we conducted a survey of canine microfilaremia in three rural provinces located on the shore of the Gulf of Thailand using a novel semi-automated microfluidic device that is easy and rapid to perform in combination with High Resolution Melting real-time PCR (HRM real-time PCR).

## 2. Materials and Methods

### 2.1. Ethics Approval

Approval number 008/2562 was acquired from the Animal Ethics Committee of the Faculty of Medicine at Siriraj Hospital, Mahidol University, based on the Ethics of Animal Experimentation of the National Research Council of Thailand. Dogs were examined with the consent of their owner.

### 2.2. Study Area

**Figure 1:**
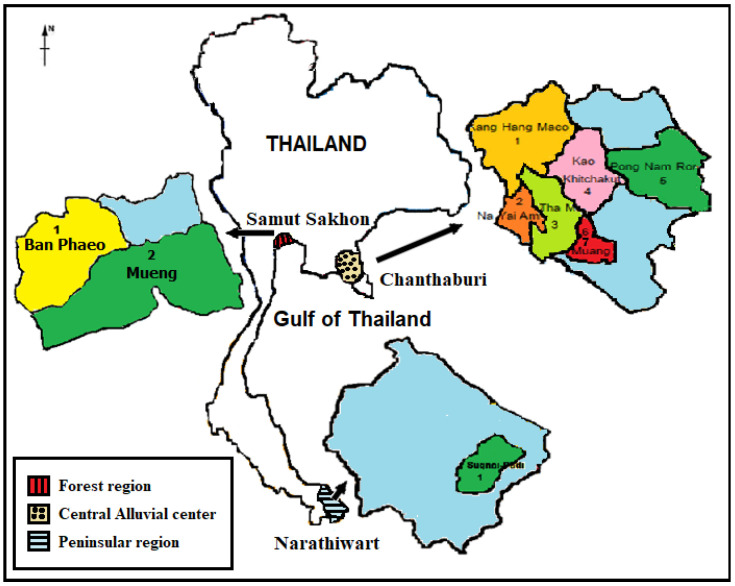
The map of Samut Sakhon, Chanthaburi, and Narathiwat provinces showing the location of the study sites.

The study was performed in Chanthaburi, Samut Sakhon, and Narathiwat provinces on the shore of the Gulf of Thailand (Figure 1) from April to December 2019. For Chanthaburi, blood samples were obtained from dogs presented for medical consultation at the veterinary clinic of one animal hospital in the capital district, as well as dogs presented for a sterilization/anti-rabies vaccination, which was conducted at the district hall of each district by the Chanthaburi Provincial Livestock Office. For Samut Sakhon, blood samples were obtained from dogs presented for sterilization/vaccination at Soi Dog foundation’s mobile veterinary clinic. For Narathiwat province, the survey was organized by the Filariasis Project, Pikhunthong Royal Development Study Center, in the Su-ngai Padi district, which is an endemic area of the nocturnally sub-periodic *B. malayi*.

### 2.3. Study Population

The study population consists of 278 dogs from Chantaburi, 217 from Samut Sakhon, and 273 dogs from Narathiwat. Dogs less than 6-months of age, pregnant dogs, and dogs whose owners did not give permission were excluded from the study. The dogs have their ages recorded from the owners’ accounts or from estimation based on body size, appearance, and dentition. The samples—one milliliter of blood—were collected from the cephalic vein and then transported to the Laboratory of the Department of Parasitology, Faculty of Medicine at Siriraj Hospital, Mahidol University for microfilariae detection and species identification.

### 2.4. Microfilariae Detection Using Microfluidic Device

**Figure 2:**
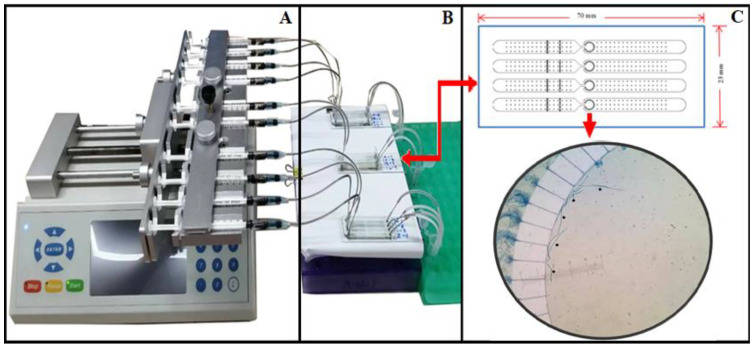
The microfluidic device for detection of microfilariae in canine blood samples. (**A**) The device consists of a sample injector, which includes an infusion pump (**B**) with 10 syringes with blunt-ended needle connected to the inlet of the microfluidic chips (**C**) The schematics of the microfluidic chips and the mf in the detection zone of the microfluidic chip.

A microfluidic device (Figure 2) was used for microfilaria detection, as described previously [16]. After suspending 50 μL of heparinized blood into 150 μL of the specifically-formulated lysis buffer A, the sample was mixed and incubated at room temperature for 10 min. Then, the sample, drawn along with 15 μL of buffer B, was put into a 1-mL syringe with a specially designed, 5-mm long blunt end needle, connecting the syringe to the adapter as well as the inlet port. The pump was switched on and the sample solution was injected into the microfluidic device via the inlet port. The microfluidic chip trapped the microfilariae while allowing the remaining solution to exit the area via the outlet port into the waste tube. The trapped mf in the microfluidic chip were further processed for species identification by Real-time PCR.

### 2.5. Species Identification of the Trapped Microfilariae by Real-Time PCR with HRM Analysis

**Figure 3:**
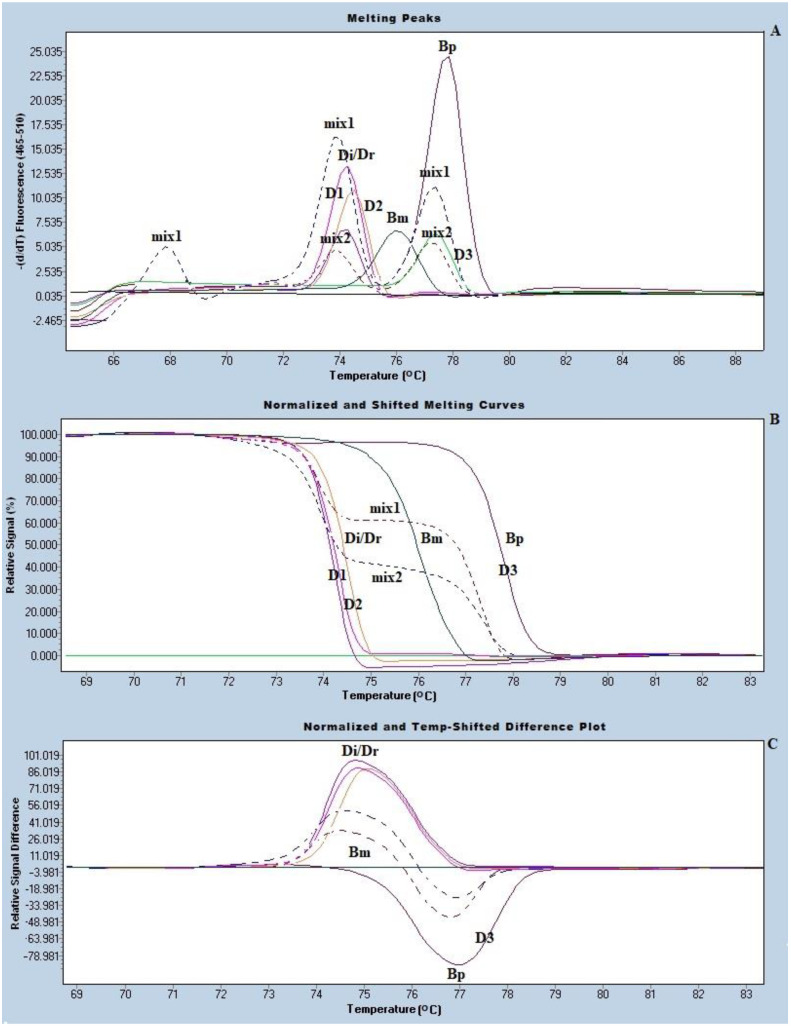
The real time PCR with High Resolution Melting (HRM) analysis for species identification of the trapped mf, (**A**) melting peaks, (**B**) normalized difference curves, and (**C**) the normalized and temperature shifted difference plot of the amplified product of a positive control, i.e., *B. malayi*, *B. pahangi*, and *D immitis*/*D. repens* and the mf positive dog samples. D1−3 representative DNAs from 119 microfilaria-positive dog blood samples. mix1 and mix2 represented mix infections (represented with a dot line).

The species of the trapped microfilariae (mf) were identified using real-time PCR with HRM analysis, as previously described [17]. The primer used based on the mitochondrial partial 12S rRNA genes of *B. malayi*, *B. pahangi*, and *D. Immitis*/*D. repen* (forward, 5′-TTTAAACCGAAAAAATATTGACTGAC, and reverse, 5′-AAAAACTAAACAATCATACATGTGCC). The condition of the real-time PCR with HRM analysis was as follows. Inject 100-μL Tris-EDTA buffer into the chip via the inlet port. Then, place the chip on a hot plate of 56 °C for 15 min. The solution containing the trapped mf was taken out through the outlet port and transferred into a 1.5-mL Eppendorf tube. After centrifugation at 15,520× g for 10 min, we discard the supernatant. The DNA was then extracted from the pelleted material using the Roche high-pure PCR template preparation kit, according to the manufacturer’s instructions (Roche Diagnostics GmbH, Penzberg, Germany). The DNA concentration was determined using a Nano Drop (Thermo Fisher Scientific, Waltham, MA, USA), and the HRM real-time PCR assay was performed on a Light-Cycler LC480 instrument (Roche, Penzberg, Germany) with primers reported previously [16,17]. The DNA from *B. malayi*, *B. pahangi*, and *D. immitis*/*D. repens* were used as positive controls (Figure 3). The LightCycler 480 gene scanning software (Roche) was implemented to construct melting curves, which were normalized, temperature-shifted, and converted to different plots.

### 2.6. DNA Sequencing

*D. immitis* and *D. repens* cannot be clearly differentiated by real-time PCR with HRM analysis using the primer based on the mitochondrial partial 12S rRNA genes due to its very closed melting temperature. It needed further confirmation. The DNA of the samples identified as *D. immitis*/*D. repens* by HRM analysis were further amplified by conventional PCR using the primers targeting Cytochrome Oxidase subunit I gene (*COXI*) (GenBank accession number AJ271614, positions 157–365) (Rishniw et al., 2006) [18]. The sequences of the forward and reverse primers were 5′ AGTGTTGATGGTCAACCTGAATTA and 5′ GCCAAAACAGGAACAGATAAAACT, respectively. The PCR products were further purified using a High Pure PCR Product Purification kit (Roche, Germany) before being submitted for DNA sequencing targeting *COXI*. The sequence alignment and analysis were performed using Clustal W [19].

## 3. Results

A total of 768 dogs were screened for microfilaremia (Table 1). The overall prevalence of microfilaraemia among dogs was 15.5%. The prevalence of microfilaremia in Chanthaburi was 16.2% (45/278), Samut Sakhon was 5.5% (12/217), and, in Narathiwat, it was 22.7% (67/273). The most common species of filariae identified in Chanthaburi was *B. pahangi*, which is a lymphatic filaria of mammals, while *D. immitis*, which is a dog heart worm, was the only species of filaria found in the dogs from Narathiwat. The age distribution of filarial infection is shown in Table 2. Dogs aged 2–5 years old showed the highest prevalence of filarial infection while dogs less than 1 year old had the lowest prevalence. The species of the trapped microfilariae in the microfluidic chamber were identified by real-time PCR with HRM analysis, as shown in Figure 3. To further confirm the species of *D. immitis/D. repens*, PCR was performed targeting the sequence of the Cytochrome Oxidase subunit I gene (*COXI*) gene. The PCR product of these samples were ≥98% identical to the reference sequences of *D. immitis*.

## 4. Discussion

We surveyed the prevalence of canine microfilaraemia in endemic and non-endemic areas of sub-periodic brugian filariasis using the microfluidic device. The device has the mf detection limit (sensitivity) of ≥20 mf per one-milliliter blood sample, which is equal to the mf detection limit of the classical technique known as thick blood smear staining. Thick blood smear staining used for microfilariae detection is time-consuming (>24 h) and the loss of microfilariae from the slide during the slide preparation process has been reported [20,21]. Observation of a large number of thick blood smear staining slides under a microscope proves an arduous task. Eye fatigue and dizziness affect many operators during their tasks, which can reduce the efficacy and sensitivity of the technique. The novel, semi-automated, microfluidic device for detection of microfilariae parasites functioned by having the microfluidic channels within the device facilitate the sieve-like sorting. Furthermore, the infusion pump enabled us to perform a larger number of blood samples and obtain results within one hour. Moreover, the instrument can reduce false negative results due to human error [16]. In terms of the advantages of real-time PCR with HRM analysis, i.e., it used only a single pair of primers with no specific probes required, a set of 384 samples can be performed in a single run and without the need for a downstream follow-up PCR step [22,23]. Moreover, the assay can detect mix infection of different filaria species. Nevertheless, application of the microfluidic device in combination with real-time HRM analysis has some difficulty for *D. immitis* and *D. repens*. It required further distinguishment using the sequencing. To obviate this concern, we can perform a thick blood smear staining technique in the mf positive blood samples identified as *D. imitis*/*D. repens* since morphology of the microfilaria of *D. immitis* and *D. repens* is clearly distinguished [24].

Recently, the first case report of *B. malayi* presented with a periorbital nodule that has occurred in a disease non-endemic area of Thailand with possibly a zoonotic origin. The nematode species was identified as *B. malayi* by histology staining and DNA sequencing of the partial mitochondrial 12S rRNA gene [25]. Another case was a 64-year-old Thai woman presented with a small nodule in her right breast without other symptoms. The nodule was detected by an imaging study and a core needle biopsy was performed, revealing a filarial-like nematode. After the molecular identification of partial mt 12rRNA gene and ITS1, the nematode was identified as *B. pahangi*, which involves a zoonotic lymphatic filaria of animals including cats and dogs [26]. These reports raise serious concern regarding the zoonotic transmission of filariasis. Thus, the high prevalence of canine microfilaraemia in Narathiwat and Chanthaburi demonstrates their importance as a reservoir host for zoonotic filariae.

No microfilariae of *B. pahangi* was detected from dogs in Narathiwat. *Mansonia* spp., which is the natural vector of *B. pahangi* and *B. malayi*, have been found in the “Toh Daeng,” which is a large swamp forest of Narathiwat [27,28,29,30]. *B. pahangi* infection has not been reported in cats or dogs in this province, which implied that *B. pahangi* infection is not endemic in Narathiwat. Furthermore, no *B. malayi* microfilariae were detected from the survey dogs.

Populations in Narathiwat are predominantly Muslims (82% Muslims and 17.9% Buddhists), and the majority of the Muslim’s households favor cats as a family pet [30]. The present study is conducted in the villages surrounding the Phru Toh Daeng swamp forest, where the majority of the inhabitants is Muslim. The highest prevalence of *B. malayi* infected cats (3.3%) has been reported from the villages, which coincided with the distribution level of *B. malayi* infected people at the village [31]. Dogs, however, are not preferred as a family pet and, therefore, unlike cats, would have limited opportunity for zoonotic transmission of *B. malayi* from human.

*B. pahangi* were identified in dogs from Samut Sakhon and was the dominant species in Chanthaburi. Two cases of brugian filariasis were reported in 2013 and 2015 in the Rayong province, the neighboring province of Chanthaburi, and *B. pahangi* from dogs was considered the likely source of infection [10]. Chanthaburi is on the eastern shore of the Gulf of Thailand, and is mostly coastal alluvial plains with a mountainous interior [32]. Larvae of *Aedes pseudotaeniatus* as well as *Ae. togoi* have been collected from Chanthaburi [11,33]. *Aedes togoi* was highly susceptible to rural strains of nocturnally subperiodic *B. malayi*, *B. pahangi*, and *D. Immitis* [11,34]. Thus, it is not surprising that *B. pahangi* was the major filarial species infecting dogs in our study.

We observed a low prevalence of canine microfilaremia in Samut Sakhon (5.5%). The Soi Dog foundation has conducted daily mobile spay and neuter clinics across the Bangkok metropolitan area for many years [35]. In 2019, the Soi Dog foundation conducted a mobile veterinary clinic in the two districts of Samut Sakhon where our study was conducted. The dogs were treated with parasiticidal drugs during the spay/neuter campaign and this likely resulted in the low prevalence of microfilaremia in the study dogs.

## 5. Conclusions

In conclusion, the prevalence of canine microfilaremia and the type of the filarial parasites reported in our study provides useful insight on the risk of zoonotic transmission. Co-habitation of infected animals and humans is an important risk factor for zoonotic filarial transmission. As observed in Samut Sakhon, implementation of the veterinary control programs can reduce animal exposure to infection. Further studies on the role of mosquito species as vectors for zoonotic filarial infection are needed since mosquito species may differ in their blood feeding patterns and host preferences. Finally, the semi-automated, microfluidic device facilitates high-throughput processing for a filarial surveillance study.

## Figures and Tables

**Table 1:** Data of microfilariae positive (%) samples detected from 768 dogs stay in Chanthaburi, Samut Sakhon, and Narathiwat provinces, Thailand. Bp = *B. pahangi*, Di = *D. immitis*, Mix Bp + Di = mix infection with *B. pahangi* and *D. immitis*, mf = microfilaria.

Study Site	Total Number of Samples (%)	mf Positive (%)	Species of mf		
			Bp (%)	Di (%)	Mix Bp + Di (%)
Chanthaburi	278 (100)	45 (162)	34 (12.25)	7 (2.5)	4 (1.46)
Samut Sakhon	217 (100)	12 (5.5)	5 (2.3)	6 (2.7)	1 (0.5)
Narathiwat	273 (100)	62 (22.7)	0 (0)	62 (22.7)	0 (0)
Total	768 (100)	119 (15.5)	39 (5.1)	75 (9.)	5 (0.7)

**Table 2:** Age distribution of 768 dogs stay in Chanthaburi, Samut Sakhon, and Narathiwat provinces, Thailand as a study population for microfilariae detection.

Study Site	Age in Years					
	≤1		2–5		>5	
	Total Number of Samples (%)	mf Positive (%)	Total Number of Samples (%)	mf Positive (%)	Total Number of Samples (%)	mf Positive (%)
Chanthaburi	66 (100)	3 (1.9)	177 (100)	39 (22)	35 (100)	3 (8.6)
Samut Sakhon	31 (100)	0 (0)	169 (100)	12 (7.1)	17 (100)	0 (0)
Narathiwat	63 (100)	0 (0)	192 (100)	57 (29.7)	18 (100)	5 (27)
Total (%)	160 (100)	3 (1.9)	538 (100)	108 (20)	70 (100)	8 (11.4)

## Data Availability

Data sharing not applicable.
